# Contraceptive use among lactating women in Ganta-Afeshum District, Eastern Tigray, Northern Ethiopia, 2015: a cross sectional study

**DOI:** 10.1186/s12884-017-1613-0

**Published:** 2017-12-16

**Authors:** Alem Gebremariam, Hadush Gebremariam

**Affiliations:** Department of Public Health, College of Medicine and Health Science, Adigrat University, Adigrat, Ethiopia

**Keywords:** Contraceptive use, Postpartum, Mode of delivery, Adigrat, Tigray

## Abstract

**Background:**

Women who are not exclusively breastfeeding are at risk of pregnancy after four to six weeks of childbirth. Postpartum **c**ontraceptive use is crucial to prevent unintended pregnancy, and to have spaced births. The study was conducted to determine the magnitude of modern contraceptive utilization and factors associated with it among lactating women in Ganta-Afeshum district.

**Methods:**

A community based cross sectional study was conducted among lactating women with children in the age group of six to twelve months. A total of 605 women were included in the study. The study participants were selected using cluster sampling method. Data were collected using structured interviewer administered Tigrigna version questionnaire. Data were analyzed using SPSS version 21. Multivariable logistic regression was used to control the effect of confounders.

**Results:**

The magnitude of institutional delivery was 96.5%. The mode of delivery of the participants was spontaneous, instrumental and caesarean section, 95.5%, 2.0%, and 2.5%, respectively. The magnitude of modern contraceptive (MC) utilization was 68.1% (95% CI: 64.4–71.8). The contraceptive method mix was dominated by Depo-Provera (58.8%) followed by implants (31.8%). Almost all the study participants had at least one antenatal care (ANC) visit (99.7%) during the pregnancy of their index child. Participants who had radio and those who delivered their recent child by assisted delivery had higher odds of modern contraceptive use.

**Conclusions:**

The magnitude of contraceptive utilization among lactating mothers in the study area was higher than the national survey reports. However, significant numbers of women are not using contraceptives in their postpartum period, making them at risk of pregnancy. Mode of delivery of the women and having radio at home were significantly associated with the women’s contraceptive utilization. Family planning information dissemination using radio in rural settings should be encouraged to increase the uptake of contraceptives in the lactating women.

## Background

Spaced birth is crucial both for the mother and the child’s health [1–3]. Birth spacing at least by two years decrease the rate of infant mortality by 50 % [2, 4]. Lactating women are at risk for unwanted or unplanned pregnancy immediately after birth. Moreover, more than 50 % (57.6%) of the women have short birth interval [5]. Therefore, contraceptive use as early as possible after child birth is important [1, 6].

The rate of contraceptive utilization is increasing from time to time [7, 8]. Similarly, in Ethiopia, the rate of contraceptive utilization has been increasing over the last decades. Over the last sixteen years, the rate of contraceptive utilization has increased from 8% to 36% [9]. The prevalence of current modern family planning utilization among all women in Oromia region Arsi zone [10] and Tigray region Adigrat town [11] was 65% and 51.3%, respectively. Contraceptive use is determined by knowledge and educational level of the women [12–14], age of the participants [15], quality of antenatal care [16], male involvement [17] and decision making power of the women [18]. Many studies have been conducted to assess the contraceptive utilization status of women generally [18, 19], with only one study targeted to lactating women [15]. So far, few studies have documented the magnitude of contraceptive utilization and factors associated with it among women who were within six to twelve months of their index delivery. Therefore, this study was conducted to document the magnitude of postpartum contraceptive utilization and factors associated with it. This specific study targeted on the lactating women with index child aged 6 months to one year will help programmers and policy makers design specific intervention targeted to these specific segments of women. Besides, it could serve as a baseline data for further future studies.

## Methods

### Study area and design

The study was conducted in Ganta-Afeshum district, Eastern zone of Tigray which is located around 903 km North of Ethiopia’s capital city, Addis Ababa. It is one of the rural districts of Eastern zone of Tigray. A community based cross sectional study was conducted among lactating women who have infant aged six to twelve months in 2015.

### Sample size and sampling procedure

A total of 605 lactating women with an infant aged six to twelve months age were included in the study. Sample size was computed using single population proportion formula considering 51.3% proportion of modern contraceptive use [11], 95% confidence level, 5% margin of error and 1.5 design effects. Considering 5% of non-response rate, the final sample size was 605.

Out of the 20 kebeles (the smallest administrative unit) in the district, twelve kebeles were selected randomly. Considering the selected kebeles as a cluster, all the eligible women found in the selected kebeles were included in the study. The health extension workers were responsible for identifying households with children between the ages of 6–12 months. We abstracted the lists of women with eligible children from the log book of health extension workers. Those lactating women with an infant aged six to twelve and lived at least six months in the selected kebeles were included in the interview. Eligible mothers who were severely ill or unable to respond for the questionnaire were excluded from the study.

### Data collection procedure

A structured pre-tested interviewer administered questionnaire was used. As much as possible, attempts were made to make questions suitable to the local setting. It was first prepared in English language, and later translated to Tigrigna language. Back translation to English was done to compare the consistency, and amendments were made accordingly.

Ten data collectors were recruited. Besides, four diploma holder nurses were recruited to supervise the data collection process. Both the data collectors and supervisors were trained for two days on the objectives of the study, study instruments and data collection procedure. The questionnaire was pre-tested on 5% sample in a similar population to the selected kebeles of the district. Revision of the questionnaire was made after the pretest. Supervisors were responsible to supervise, assist interviewers and collect filled questionnaires every day and check for consistencies and completeness of filled questionnaires. Questionnaires with problems were sent back to interviewers for re-interview. The investigators were responsible for coordination and supervision of the overall data collection process.

### Data analysis

The collected data were entered to Epi Info then exported to window based Statistical Package for Social Sciences (SPSS) version 21.0. Data were cleaned for inconsistencies and missing values and amendment was considered as needed. Simple frequencies were run to see the overall distribution of the study subject with the variables under study. Bivariate analysis was used to determine the association between different factors and the outcome variable.

Multivariable logistic regression was used to identify the relative importance of each independent variable to the outcome variable by controlling for the effects of other variables. Variables with *p*-value of 0.2 or less on bivariate analysis were included in the multivariable analysis. Multi-collinearity was checked using the Variance Inflation Factor (VIF), and those with VIF greater than 10 were excluded from the model. Participants’ age, their index child’s age, family size, and total number of births were treated as continuous variable. Family size was correlated with total number of births, and excluded from the multivariable model. The association between modern contraceptive use and independent variables was determined using Odds Ratio (OR) with 95% Confidence Interval (CI). The level of significance was taken at α = 0.05.

### Ethical statement

Prior to the data collection, ethical clearance was obtained from Adigrat University ethical clearance committee. Permission letter was written from Tigray Regional Health Bureau to the study district, Ganta-Afeshum. Besides, written cooperation letter was obtained from Ganta-Afeshum district health office to each of the selected kebeles. Interviewers obtained oral informed consent of the respondents prior to the interview. Participation in the study was completely voluntary and refusal to respond to some of the questions or interruption from the study was possible. All the information obtained from the respondents remained confidential and anonymous. Finally, the dissemination of the finding does not refer specific respondents but the general source population.

## Results

### Socio-demographic characteristics of the participants

A total of 599 women responded fully to the questionnaire making the response rate 99.5%. The mean age of the participants was 30.8 years (SD = 6.29). The median family sizes of the participants were 5 with a minimum of 2 and maximum of 12. Almost all (99.8%) of the participants were Tigrie by Ethnicity. Sixty-one percent of the participants were farmers. Forty-one percent of the participants were illiterate (Table 1). Seventy percent and 15 % (15.3%) of the participants have radio and television in their home, respectively.

**Table 1:** Socio-demographic characteristic of participants in Gnata-Afeshum district, Tigray, 2015 (*n* = 599)

Variables	Frequency (n)	Percent (%)
Marital status of mother		
Married	572	95.5
Ever Married	27	4.5
Religion		
Orthodox	585	97.7
Muslim	14	2.3
Participants’ level of education		
Illiterate	246	41.1
Elementary (1–4)	99	16.5
Grade 5–8	127	21.2
Grade 9 and above	127	21.2
Partners’ level of education		
Illiterate	151	25.2
Elementary (1–4)	103	17.2
Grade 5–8	164	27.4
Grade 9 and above	181	30.2
Participant’s occupation		
Farmer	240	40.0
Housewife	313	52.3
Employed^a^	46	7.7

^a^Refers to those who were merchant, daily laborer, and government and privet employed

### Participants’ maternal health service utilization

The median number of births given by the participants was 3 with the minimum of one and maximum of ten births. The magnitude of institutional delivery was 96.5%. The mode of delivery of the participants was spontaneous, instrumental and caesarean section, 95.5%, 2.0%, and 2.5%, respectively. Sixteen (2.7%) of the participants were assisted by their relatives during the birth of their index child. The magnitude of Modern Contraceptive (MC) utilization was 68.1% with 95% CI of 64.4% to 71.8%. The contraceptive method mix was dominated by Depo-Provera (58.8%) followed by implants (31.8%), and pills (4.9%) (Table 2).

**Table 2:** Reproductive history of study participants’ of Ganta-Afeshum district, Tigray, 2015 (*n* = 599)

Variables	Frequency (n)	Percent (%)
History of abortion		
Yes	79	13.2
No	520	86.8
History of still birth		
Yes	33	5.5
No	566	94.5
Current modern contraceptive use status		
Yes	408	68.1
No	191	31.9
Type of MC used		
Depo-Provera	240	58.8
Implants	130	31.8
Pills	20	4.9
Intra uterine contraceptive device	14	3.4
Female sterilization	4	0.1
Number of ANC visit		
1	4	0.7%
2	19	3.2%
3	132	22.1%
4	380	63.7%
> = 5	62	10.3%
Birth assisted by		
Health provider	576	96.2
Health extension worker	4	0.7
Traditional birth attendant	3	0.5
Family relatives	16	2.7
Mode of delivery		
Spontaneous vaginal delivery	572	95.5
Assisted delivery	12	2.0
Cesarean delivery	15	2.5

*MC* modern contraceptives, *ANC* Antenatal care

Almost all the study participants had at least one Antenatal Care (ANC) visit (99.7%) during the pregnancy of their index child. Three fourths of the participants had made four or more ANC visits (Table 2). During their ANC visit, participants remembered as they received advice on place of delivery (81.6%), breastfeeding (56.6%), child immunization (39.4%), mother’s tetanus toxoid vaccination (49.9%) and family planning utilization after birth (37.6%) by their care givers.

### Factors associated with modern contraceptive use

On bivariate analysis, sex of their index child, marital status, family size, partners’ education status, having radio, number of births and mode of delivery were found significantly associated with modern contraceptive use (Table 3). After inclusion of the variables with *p*-value of less than or equal to 0.2 in to the multivariable logistic regression model, having a radio and mode of delivery were found statistically significantly associated with modern contraceptive use (Table 4).

**Table 3:** Bivariate analysis of socio-demographic and reproductive characteristics of the participants with modern contraceptive utilization

Variables	MC Use		Crude OR(95% CI)	*P*-value
	Yes, n(%)	No, n(%)		
Sex of the child				
Male	246(71.5%)	98(28.5%)	Ref.	
Female	162(63.5%)	93(36.5%)	0.69(0.49, 0.98)	0.039
Index child’s age	408(68.1%)	191(31.9%)	0.95(0.86, 1.04)	0.263
Age of the participants	408(68.1%)	191(31.9%)	0.98(0.95, 1.00)	0.154
Mother’s marital status				
Married	394(68.9%)	178(31.1%)	2.05(0.95, 4.46)	0.069
Ever Married	14(51.9%)	13(48.1%)	Ref.	
Family size	408(68.1%)	191(31.9%)	0.91(0.83, 0.99)	0.030
Participants’ level of education				
Illiterate	165(67.1%)	81(32.9%)	Ref.	
Elementary (1–4)	63(63.6%)	36(28.3%)	0.85(0.52, 1.402)	0.54
Grade 5–8	91(71.7%)	36(28.3%)	1.24(0.77, 1.98)	0.367
Grade 9 and above	89(70.1%)	38(29.9%)	1.15(0.72, 1.83)	0.555
Partners’ level of education				
Illiterate	106(70.2%)	45(29.8%)	Ref.	
Elementary (1–4)	60(58.3%)	43(41.7%)	0.59(0.35, 1.00)	0.050
Grade 5–8	101(61.6%)	63(38.4%)	0.68(0.42, 1.08)	0.108
Grade 9 and above	141(77.9%)	40(22.1%)	1.49(0.91, 2.45)	0.110
Mother’s occupation				
Farmer	157(65.4%)	83(34.6%)	Ref.	
Housewife	217(69.3%)	96(30.7%)	1.19(0.83, 1.71)	0.330
Employed	34(73.9%)	12(26.1%)	1.49(0.73, 3.04)	0.265
Have radio				
Yes	304(72.4%)	116(27.6%)	Ref.	
No	104(58.1%)	75(41.9%)	0.52(0.36, 0.76)	0.001
Have TV				
Yes	56(62.2%)	34(37.8%)	Ref.	
No	352(69.2%)	157(30.8%)	1.36(0.85, 2.17)	0.194
Total number of births	408(68.1%)	191(31.9%)	0.89(0.82, 0.97)	0.009
History of abortion				
Yes	49(62.0%)	30(38.0%)	Ref.	
No	359(69.0%)	161(31.0%)	1.36(0.83, 2.23)	0.214
History of still birth				
Yes	20(60.6%)	13(39.4%)	Ref.	
No	388(68.6%)	178(31.4%)	1.41(0.68, 2.91)	0.343
Number of ANC visit	408(68.1%)	191(31.9%)	1.06(0.83, 1.35)	0.603
Advice on family planning during ANC				
Yes	245(65.9%)	127(34.1%)	1.36(0.95, 1.96)	0.094
No	163(72.4%)	62(27.6%)	Ref.	
Place of delivery				
Home	16(76.2%)	5(23.8%)	Ref.	
Health institution	392(67.8%)	186(32.2%)	0.65(0.23, 1.83))	0.422
Mode of delivery				
Spontaneous vaginal delivery	384(67.1%)	188(32.9%)	0.25(0.07, 0.86)	0.027
Non-spontaneous delivery	24(88.9%)	3(11.1%)	Ref.

**Table 4:** Multivariable analysis of selected variables of the participants with modern contraceptive utilization

Variables	COR(95% CI)	AOR (95% CI)	*P*-Value
Sex of the index child			
Male	Ref.	Ref.	
Female	0.69(0.49, 0.98)	0.74(0.51, 1.07)	0.109
Age of the participants	0.98(0.95, 1.00)	1.02(0.97, 1.06)	0.433
Participant’s marital status			
Married	2.05(0.95, 4.46)	1.99(0.88, 4.51)	0.097
Ever married	Ref.	Ref.	
Partner’s level of education			
Illiterate	Ref.	Ref.	
Elementary (1–4)	0.59(0.35, 1.00)	0.58(0.33, 1.00)	0.053
Grade 5–8	0.68(0.42, 1.08)	0.63(0.37, 1.07)	0.087
Grade 9 and above	1.49(0.91, 2.45)	1.28(0.70, 2.31)	0.415
Have radio			
Yes	Ref.	Ref.	
No	0.52(0.36, 0.76)	0.52(0.35, 0.77)	0.001
Have Television			
Yes	Ref.	Ref.	
No	1.36(0.85, 2.17)	1.55(0.93, 2.57)	0.086
Total number of births	1.11(1.03, 1.21)	0.89(0.78, 1.02)	0.096
Advice on family planning during ANC			
Yes	1.36(0.95, 1.96)	1.46(0.99, 2.15)	0.051
No	Ref.	Ref.	
Mode of delivery			
Spontaneous vaginal delivery	0.25(0.07, 0.86)	0.27(0.08, 0.94)	0.039
Non-spontaneous delivery	Ref.	Ref.	

Women who had no radio in their home had 48% lower odds of modern contraceptive use compared to those who had radio in their home (AOR = 0.52; 95% CI: 0.35, 0.77). Similarly, mothers whose recent mode of delivery was spontaneous vaginal delivery had 75% lower odds of modern contraceptive use compared to those whose mode of delivery was assisted vaginal delivery and caesarean delivery (AOR = 0.25; 95% CI: 0.07, 0.86). Though it was marginally significant (AOR = 1.46; 95% CI: 0.99, 2.15), participants who had received advice on family planning during their antenatal care had higher odds of contraceptive use during their postpartum period (Table 4).

## Discussion

This community based cross-sectional study was attempted to assess the magnitude of modern contraceptive utilization among lactating mothers in Gant-Afeshum district. The risk of pregnancy increases when a woman shifted from the period of exclusive breastfeeding to the period of nearly fully breastfeeding. Therefore, using contraceptives is a guarantee to prevent unwanted pregnancy [1]. The magnitude of modern contraceptive utilization rate among lactating women was 68.1% (95%CI: 64.4%, 71.8%). This is higher than the recent national demographic health survey report of modern contraceptive utilization rate [9], and study conducted in Gondar, Northwest Ethiopia [15]. The difference could be explained by the difference in the study subjects where our study is targeted to women who were with index child aged 6 months to one year. Whereas the study in Gondar was inclusive of all women who give birth within one year of birth which included women in the exclusive breastfeeding. Besides, previous study conducted in the neighboring district reported 51.3% utilization rate among all women [11]. Moreover, this finding is comparable with the findings documented in Arsi Zone [10], and Dawro zone, Southern Ethiopia [18] which was 65%, and 72.8%, respectively. However, it is lower than the finding in Malawi where 75% of the lactating women were utilizing contraceptives [20]. Similar to other studies [12, 21, 22], the dominant method used was Depo-Provera. This is explained by the simplicity of the procedure to use and to keep secret of their partner [14].

Having radio, and mode of delivery were associated with postpartum contraceptive utilization. Those who had no radio had 48% lower odds of contraceptive utilization. The study area is a rural district where electricity access is poor and it is difficult to have television. But they can use radio and help them to get family planning related information disseminated using the radio. This is not uncommon in other studies where health providers and mass medias take the highest share of family planning information [12, 23–25]. Similarly, in Uganda, exposure to media (OR = 1.30; 95% CI = 1.05–1.61) was significantly associated with postpartum contraceptive utilization [26].

During the antenatal care, postpartum family planning is one of the counseling given to the women about their pregnancy care. Though it is marginally significant, women who had received family planning advice during their pregnancy care had 46% higher odds of contraceptive use. This was also documented in Gondar [15], Kenya and Zambia [16] where the intensity of antenatal care was associated with postpartum contraceptive use.

Women whose mode of delivery was spontaneous had a 73% lower risk of contraceptive utilization. This could, be explained by the difference in the health professionals who attended the labor. Moreover, women who delivered by cesarean section or assisted delivery could be at higher fear of next pregnancy and want to avoid pregnancy. Mengesha and his colleagues also documented higher odds of contraceptive use among women who delivered with the assistance of skilled attendant (OR = 1.88) compared to those who did not receive skilled care during delivery [22]. Similarly, a study in Uganda, found that deliveries attended by skilled birth attendance (OR = 1.39; 95% CI = 1.12–1.17) and 1–2 days timing of post-delivery care (OR = 1.68; 95% CI = 1.14–2.47) were associated with postpartum contraceptive utilization [26].

Postpartum contraceptive utilization was associated with primary or higher education, age of woman, and number of surviving children [26]. However, this study did not find statistical significant difference across these characteristics. Similar to our finding, partner’s level of education was not significantly associated with postpartum contraceptive use in Gondar [22]. Though it is not statistically significant, the multivariable model result suggested an increase in odds of contraceptive use as the level of partner’s education increased. This insignificance could be explained by the sample size we used which may not be sufficient enough to detect the difference of contraceptive use across the levels of partner’s education.

The cross-sectional nature of the data has causality challenges. It is difficult to ascertain the association between postpartum contraceptive utilization and the predictor variables since they were measured at one point in time. The study did not address all health system related factors and history of prior use of contraceptives in previous pregnancies that could affect the current postpartum family planning utilization. Though training on the objectives and procedures of the data collection was given to the data collectors, the issue of interviewer and social desirability bias is inevitable.

## Conclusions

Compared to the national and pocket study findings, the magnitude of contraceptive utilization among lactating mothers in the study area was higher than the national survey findings. However, significant numbers of women were not using contraceptives in their postpartum period, making themselves at risk of pregnancy. Family planning advice during antenatal care, mode of delivery of the women and having a radio were significantly associated with the women’s postpartum contraceptive utilization. Family planning information dissemination using radio in the rural settings should be encouraged to increase the uptake of contraceptives in the lactating women. Strengthening of the family planning advice provision to the women during their antenatal care should be emphasized. Detail prospective studies should be conducted to investigate the effect of antenatal care service utilization on postpartum contraceptive utilization.

## Abbreviations

ANC: Antenatal Care
AOR: Adjusted Odds Ratio
CI: Confidence Interval
COR: Crude Oddis Ratio
MC: Modern Contraceptive
OR: Odds Ratio
